# Psychometric Properties of Quality of Life Questionnaires for Patients with Breast Cancer-Related Lymphedema: A Systematic Review

**DOI:** 10.3390/ijerph19052519

**Published:** 2022-02-22

**Authors:** Estu Meilani, Asfarina Zanudin, Nor Azlin Mohd Nordin

**Affiliations:** Physiotherapy Program, Center for Rehabilitation and Special Needs Studies, Faculty of Health Sciences, Universiti Kebangsaan Malaysia, Kuala Lumpur 50300, Malaysia; p104866@siswa.ukm.edu.my (E.M.); norazlin8@ukm.edu.my (N.A.M.N.)

**Keywords:** breast cancer-related lymphedema, psychometric properties, quality of life, questionnaire

## Abstract

Backgrounds: Assessing quality of life (QoL) using a well-developed and validated questionnaire is an essential part of a breast cancer-related lymphedema (BCRL) treatment. However, a QoL questionnaire with the best psychometric properties is so far unknown. The aim of this systematic review is to evaluate the psychometric properties of the questionnaires measuring the QoL of patients with BCRL. Methods: A thorough search was performed to identify published studies in electronic databases such as Medline (via Ovid), EBSCOhost, PubMed, Scopus, and Web of Science, on 8 February 2022, by using search terms as follows: ‘quality of life’; ‘breast cancer’; ‘upper limb’; ‘lymphedema’; ‘questionnaire’; and ‘measurement properties.’ Two reviewers conducted article selection, data extraction, and quality assessment independently. The third reviewer helped solve any possible disagreements between the two reviewers. The COSMIN checklist and manual were used to assess the quality of included studies. Results: A total of nineteen articles with nine questionnaires were included and assessed using the COSMIN Risk of Bias checklist. Most studies only assessed content validity, structural validity, internal consistency, reliability, and construct validity. Lymph-ICF-UL showed the most ‘sufficient’ and ‘high’ quality of evidence ratings for its measurement properties. Conclusion: The most appropriate questionnaire for use based on our assessment is Lymph-ICF-UL.

## 1. Introduction

Breast cancer is the most prevalent cancer diagnosis in developed and less developed countries worldwide. It impacts over two million women each year and causes the most considerable number of cancer-related deaths among women. According to the International Agency for Research in Cancer, more than six hundred thousand women globally died from breast cancer in 2018 [1]. In recent years, the advancement of breast cancer management has led to a higher survival rate from this disease [1], resulting in greater demand for post-cancer care [2].

However, these advanced improvements also come with side effects, such as fatigue, psychological distress, arm lymphedema, or sexual dysfunction [3,4,5]. Arm lymphedema or breast cancer related-lymphedema (BCRL) affects almost one in five breast cancer survivors (21.4%) [6], with the overall incidence rate ranging from 15.5% to 54% [6,7,8,9,10]. The incidence is most likely to increase over time, up to 24 months following a breast cancer diagnosis or surgery [6]. Lymphedema is a chronic swelling resulting from a protein-rich fluid over-accumulation in extracellular space due to the transport capacity insufficiency of the lymphatic system [11,12]. Based on its etiology, there are two types of lymphedema: primary and secondary [13]. Factors that could increase the risk of developing lymphedema after breast cancer treatments are scar from the surgical procedures [14], the number of lymph nodes removed [15,16], chemotherapy [9], radiotherapy [15,16], obesity, and being married [9]. In terms of a living region, approximately one in five breast cancer survivors living in North America, Australasia, Asia, and the Middle East develop BCRL. Meanwhile, less than one in six survivors living in Europe, the United Kingdom, and South America develop lymphedema following their breast cancer treatment [6]. Moreover, having less than three children may increase the BCRL risk due to less-frequent movement of the affected side in doing the house chores and family care [17].

Swelling, pain, limited joint mobility, the thickness of skin [18], depression, anxiety, and negative body image are the most frequently reported complaints of BCRL patients [19]. Limited joint mobility, swelling, pain, and skin problems in the affected area could lead to functional impairment and increase the risk of skin infection [18,19]. These symptoms would limit the patients’ abilities to intently participate in household and work-related activities, resulting in the mitigation of their quality of life (QoL) [20,21]. Repercussions of these BCRL symptoms on patients’ daily activities must be adequately addressed to improve patients’ physical and psychological functioning and, subsequently, the overall QoL [22,23,24,25].

Given the fact that BCRL could affect the way a patient feels and functions, patient-reported outcome measures (PROMs) may help clinicians in assessing the effectiveness of BCRL treatments [26,27,28]. PROM is a standardized questionnaire that is completed by a patient to comprehensively measure their perception of their own well-being as the result of a certain condition, including BCRL [26]. Despite the importance of assessing the QoL of BCRL patients [22,23,24,25], a robustly-developed PROM with the best psychometric properties is so far unknown. To be considered as a robust instrument, a PROM should meet the standard criteria for measurement properties such as whether the PROM measures the construct it purports to measure and whether it is easily understood by the target population (validity); whether the PROM measures the same way each time and detects the changes accurately without measurement error (reliability); and how much changes are considered clinically important (responsiveness) [29,30].

Several systematic reviews of QoL questionnaires that have been [21,31,32] published previously were either: not focused on studies that only assess psychometric properties [21]; did not assess different types of lymphedema-specific questionnaires [31]; not focused on the BCRL population, but using general population and non-BCRL population [21,31]; or not using a specific checklist to assess psychometric properties, such as consensus-based standards for the selection of health measurement instruments (COSMIN) risk of bias checklist [32]. Thus, our systematic review aims to evaluate the psychometric properties of the questionnaires measuring QoL in BCRL patients using an exclusively designed COSMIN checklist. Finally, based on this review, we will propose the most suitable questionnaire for future use of QoL assessment in breast cancer-related upper limb lymphedema patients.

## 2. Materials and Methods

### 2.1. Study Protocol

The study protocol of this review was registered in the International Prospective Register of Systematic Reviews (PROSPERO) with the registration number CRD42020220119. The study protocol can be found elsewhere [33].

### 2.2. Search Strategy

The following electronic databases were searched on 8 February 2022: Medline (via Ovid), EBSCOhost, PubMed, Scopus, and Web of Science. The main terms used for the database search were: ‘quality of life’, ‘breast cancer’, ‘upper limb lymphedema’, ‘questionnaire’, and ‘measurement properties’. A few additional sensitive search and exclusion filters developed by Terwee et al. [34] were applied to each database. The details of this database search are provided in Supplementary File S1. The references list of identified articles was manually screened to find more relevant studies.

### 2.3. Study Selection

After removing the duplicates, one author (E.M.) reviewed and screened the list of identified articles based on their titles, followed by their abstracts. Full-text articles were then retrieved and examined by two authors (E.M. and A.Z.) to obtain a final list of eligible studies according to the predetermined inclusion and exclusion criteria. Any conflicting opinions throughout the study selection process were resolved by further review and discussion involving the third author (N.A.M.N.).

The following inclusion criteria were applied: (1) the study assessed one or more measurement properties as described by the COSMIN steering committee, which includes reliability (internal consistency and measurement error), validity (content validity, construct validity, and criterion validity), and responsiveness [29]; (2) the study used either an original or translated version of a lymphedema specific-questionnaire that measured the aspects of QoL, such as physical, psychological, and social well-being; (3) at least 50% of the patients included in the study were diagnosed with breast cancer-related upper limb lymphedema; and (4) full-text articles that were published in the English language from database inception up to and including the 8 February 2022. 

The studies were excluded when they only consisted of abstract, dissertation, conference proceedings, editorials, opinion pieces, review papers, letters, single case studies, short communications, or technical notes. Furthermore, studies in healthy populations and studies whose primary purpose is not to assess psychometric properties as defined above were also excluded from this review.

### 2.4. Data Extraction

All information from the included studies and questionnaire or patient-reported outcome measures (PROMs) were extracted onto a data extraction sheet. Extracted data included: (1) characteristics of PROM, such as name of the PROM, reference of the article in which the PROM was used, the country in which the PROM was evaluated, number of the items, subscales being measured, recall period, response option, scoring system, the original language of the PROM and the available translations so far; (2) characteristics of included studies of PROM assessing QoL in BCRL, including author, country, PROM being used, the objective of the study, sample size, age mean, gender, and lymphedema characteristics (type, duration, severity).

### 2.5. Quality Assessment

The quality of full-text articles identified as eligible studies was assessed using the COSMIN checklist and scoring manual. COSMIN steering committee developed an extensive methodological guideline and checklists for systematic reviews of PROMs [29]. The COSMIN guideline was well-established per the current guidelines for reviews, such as the Cochrane Handbook for systematic reviews of intervention [35] and for diagnostic test accuracy reviews [36], the Preferred Reporting Items for Systematic Reviews and Meta-Analyses (PRISMA) statement [37], the Institute of Medicine (IOM) standards for systematic reviews of comparative effectiveness research [38], and the Grading of Recommendations Assessment, Development and Evaluation (GRADE) principles [39].

We utilized the COSMIN risk of bias checklist, one of three versions of the original COSMIN checklists to assess the quality of included PROMs [30]. This checklist provided preferred design requirements and statistical methods of each measurement property. The term ‘risk of bias’ abides by the Cochrane methodology for systematic reviews of trials and diagnostic studies, which indicates whether the study’s methodological quality results are trustworthy [29]. The COSMIN risk of bias checklist consists of ten boxes for PROM development standards (box 1) and for nine measurements properties which are content validity (box 2), structural validity (box 3), internal consistency (box 4), cross-cultural validity/measurement invariance (box 5), reliability (box 6), measurement error (box 7), criterion validity (box 8), hypotheses testing for construct validity (box 9), and responsiveness (box 10) [30]. Table 1 presents the definitions of these measurement properties adapted from the COSMIN guideline [30].

Quality assessment of included PROMs was performed in three steps. Two reviewers performed the quality assessment independently (E.M. and A.Z.). A further discussion with the third reviewer (N.A.M.N.) was available if no agreement could be reached.

#### 2.5.1. Step 1. COSMIN Risk of Bias Checklist

The methodological quality assessment was performed using corresponding boxes in the COSMIN risk of bias (RoB) checklist [30]. Each box consists of 4 to 35 items and is rated with a four-point rating system which is, ‘V = very good’, ‘A = adequate’, ‘D = doubtful’, and ‘I = inadequate’. The overall rating of each study was determined by taking the lowest rating of any items within each box. This rating would be used in grading the quality of evidence (step 3b) [29].

#### 2.5.2. Step 2. Applying Criteria for Good Measurement Properties

*i*. *Step 2a: Content validity*

The result of each study on PROM development and content validity was rated against the 10 criteria for good content validity. The ratings of all available studies were then qualitatively summarized to determine whether the overall ratings of each PROM were sufficient (+), insufficient (−), or indeterminate (?) in terms of relevance, comprehensiveness, comprehensibility, and overall content validity [40]. Suppose the content validity of the PROM was rated as insufficient. In that case, the PROM should not be recommended for use and will be excluded from further evaluation of the remaining measurement properties [30].

*ii*. *Step 2b: Remaining measurement properties*

The result of each study on other measurement properties was rated against the updated criteria for good measurement properties as either sufficient (+), insufficient (−), or indeterminate (?) [29]. The updated criteria for good measurement properties are provided in Table 2.

#### 2.5.3. Step 3. Summary of Evidence

*i*. *Step 3a. Content validity*

The overall ratings of each PROM determined in step 2a were also rated for the quality of evidence as either high, moderate, low, or very low, using a modified GRADE approach. GRADE rated the quality of evidence by considering the following factors: risk of bias (quality of the studies), inconsistency (of the results of the studies), indirectness (evidence comes from different populations, interventions, or outcomes than the ones of interest in the review), imprecision (wide confidence intervals), and publication bias [39]. However, only three of these factors were relevant in evaluating content validity, including risk of bias, inconsistency, and indirectness [40].

*ii*. *Step 3b. Remaining measurement properties*

The results of all available studies were summarized and rated again against the criteria for good measurement properties (Table 2) to determine whether the measurement properties of each PROM were sufficient (+), insufficient (−), inconsistent (±), or indeterminate (?). If the results per study are all-sufficient (or all-insufficient or all-indeterminate), the overall rating will also be sufficient (or insufficient or indeterminate). In principle, to rate the qualitatively summarized results as sufficient (or insufficient), 75% of the result should fit the criteria [29]. Next, the quality of evidence of each measurement property was graded using the modified GRADE approach [39]. When evaluating the quality of measurement properties, only four of five factors were considered: risk of bias, inconsistency, imprecision, and indirectness. Meanwhile, publication bias is difficult to assess in studies on measurement properties [29].

## 3. Results

### 3.1. Study Outcomes

**Figure 1:**
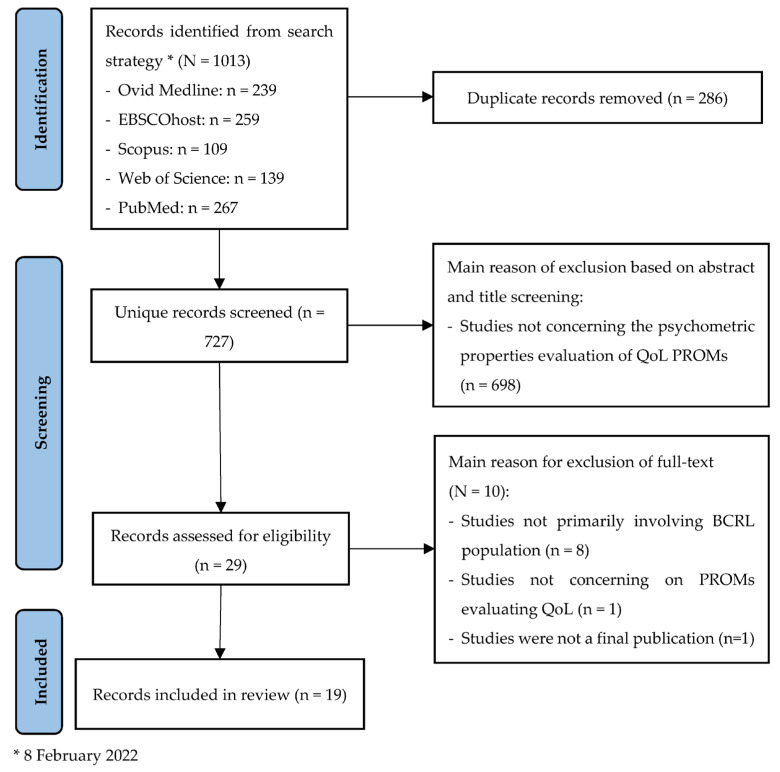
PRISMA flowchart on the study selection process.

The literature search identified 1013 articles. The details of the study selection process were provided in the PRISMA flow chart (Figure 1). After duplicates were removed, a total of 698 studies were then excluded based on the title and abstract screening. Subsequently, 29 articles were included in the full-text screening. In the full-text screening, 10 articles were excluded, and finally, a total of 19 articles met the inclusion criteria.

### 3.2. Characteristics of Included Studies

Table 3 presents the characteristics of the 19 included studies. Thirteen studies translated and validated the original questionnaire into their respective languages. One study performed a revision of a PROM and investigated its measurement properties. One study conducted an assessment on the responsiveness of a questionnaire. The remaining four studies developed a new questionnaire then validated it. The average age of the samples included in the studies ranged from 19 to 92 years old. Not all measurement properties were assessed for each PROM in the included studies. Reliability was assessed multiple times: internal consistency and test-retest reliability were assessed 18 and 14 times, respectively, while the assessment for measurement error was performed four times. All studies assessed the content validity, while the remaining validity domains were assessed 12 times for structural validity, 17 times for construct validity via hypothesis testing, and once for criterion validity. Meanwhile, responsiveness was only assessed twice.

### 3.3. Characteristics of Included PROMs

The characteristics of nine identified PROMs are presented in Table 4. All included PROMs were evaluated in various languages. The number of items ranged from 14 to 68, with total subscales or domains ranging from two to seven. Five PROMs did not provide a specific recall period; meanwhile, the recall period of the remaining four ranged from right at the moment of assessment to two weeks. All included PROMs used total scores and domains scores to determine the quality of life, except LYMPH-Q Upper Extremity that only used scales scores in determining the patient’s quality of life.

### 3.4. Quality Assessment

#### 3.4.1. Methodological Quality and Rating against Good Measurement Properties for Results of Each Included Studies

The methodological quality of 19 studies assessing psychometric properties of QoL PROMs was rated as “very good” (41 times), “adequate” (13 times), “doubtful” (21 times), and “inadequate” (11 times). Results of all the studies were rated against criteria for good measurement properties and showed 109 times for “sufficient”, four times for “indeterminate”, and nine times for “insufficient” ratings. The study findings of included studies, the methodological quality rating, and the rating against good measurement property are presented in Table 5.

#### 3.4.2. Overall Rating and Grading of the Quality of Evidence per Measurement Properties for Each PROM

Each study’s results were summarized and rated again against criteria for good measurement by COSMIN to examine each PROM’s quality as a whole. The summarized results of each PROM were rated as “sufficient” (39 times), “indeterminate” (three times), and “insufficient” (six times). The detailed assessment of the summarized results is presented in the last column of each PROM assessment in Table 5. The quality of evidence for each measurement property of each PROM is provided in Table 6.

Lymphedema Quality of Life Tool-Arm (LYMQOL-Arm) is a self-reported questionnaire designed to measure QoL in patients with BCRL. This questionnaire assesses the upper limb lymphedema symptoms and patients’ ability to perform functional daily activities. LYMQOL-Arm consists of 21 items, with the first item (“Affect daily activities”) consisting of seven sub-questions (a-h). There are three studies translating LYMQOL-Arm into the Turkish language. The three studies evaluate a different number of items, Bakar et al. and Karayurt et al. evaluated the items without including the seven sub-questions into their assessment (21 items) [41,42]. Meanwhile, the other one, Borman et al. included all the seven sub-questions into their analysis, resulting in a total of 28 items assessed [43]. All Turkish versions of LYMQOL-Arm were rated “sufficient” for content validity and construct validity [41,42,43]. However, LYMQOL-Arm B was rated “insufficient” for structural validity because the model fit indices of the confirmatory factor analysis (CFA) did not meet the criteria for good measurement properties (CFI and TLI <0.95; RMSEA >0.06). Due to this “insufficient” rating for structural validity, internal consistency for LYMQOL-Arm B was rated “indeterminate”, even though the Cronbach’s α values of both domains and overall scores were good to excellent. Moreover, LYMQOL-Arm B was also rated “insufficient” for reliability because the ICC values were less than 0.7 [43]. Both versions’ quality of evidence for content validity was “low”. The low rating was given due to the lack of information on the content validation process [41,42,43]. LYMQOL-Arm A was rated “low” for reliability due to a low sample size (<100) and only one study with “adequate” quality available [42,43]. LYMQOL-Arm B received a “very low” rating for structural validity because it only has one study with “inadequate” quality [43].

Lymphedema Life Impact Scale version 1 (LLIS ver.1) is an 18-item self-reported questionnaire that measures physical, psycho-social, and functional impact on the lives of patients with BCRL. Each item is rated on a five-point Likert scale ranging from 1 to 5. LLIS ver.1 was rated “sufficient” for content validity, internal consistency, reliability, and construct validity with “moderate” quality of evidence. The “moderate” rating was given because some of the study population was not BCRL patients (8.7% of the total study population for structural validity and internal consistency, 22.65% of the total study population for reliability, and 2.8% of the total study population for construct validity, were lower limb lymphedema patients) [44,45].

Lymphedema Life Impact Scale version 2 (LLIS ver.2) is the updated version of LLIS ver.1 that included a question regarding knowledge of lymphedema management and used a 0 to 4 scoring system. LLIS ver.2 also has a separate question regarding the number of infection occurrences. It was rated “sufficient” for content validity, structural validity, internal consistency, reliability, and construct validity. However, LLIS ver.2 was rated “insufficient” for criterion validity due to weak correlation with the gold measurement standard limb volume differences (r < 0.40, *p* < 0.05). LLIS ver.2 was rated “high” only for construct validity. Meanwhile, the quality of evidence of the other measurement properties was varied from “very low” for reliability, “low” for content validity and structural validity, to “moderate” for internal consistency and criterion validity These scores were given due to the following reasons: a poor description of content validation process; only one available study with “adequate” quality on structural validity and reliability; the insufficient sample size (<50 for reliability; <100 for criterion validity); and also because the study included non-lymphedema patients for structural validity, internal consistency, criterion validity analysis (44.8% of the total study population) [46,47].Lymphedema Functioning, Disability, and Health Questionnaire for Upper Limb (Lymph-ICF-UL) is a 29-item self-reported questionnaire developed by Devoogdt et al. in 2011 that aimed to quantitatively evaluate problems in functioning related to lymphedema of the upper limb [49]. When compared to the other included PROMs, Lymph-ICF-UL assessed the greatest number of measurement properties as recommended by COSMIN. It was rated “sufficient” for all reported measurement properties. Lymph-ICF-UL received a “high” quality of evidence score for all reported measurement properties, except structural validity and responsiveness which rated moderate; and measurement error which scored “low” due to an insufficient number of at least “adequate” quality studies [48,49,50,51,52,53].

Lymphedema Symptom Intensity and Distress Survey-Arm (LSIDS-A) is a lymphedema-specific questionnaire that assesses upper limb lymphedema and its multidimensional symptoms. LSIDS-A was rated as “sufficient” for all reported measurement properties, except “insufficient” on construct validity because more than 25% of study results were not aligned with the predetermined hypotheses. The quality of evidence of LSIDS-A was scored “very low” on reliability because there was an insufficient sample size (<100) and only one “doubtful” quality study available. Moreover, the content validity was scored “low” due to the lack of information in the content validation process [54,55].

Upper Limb Lymphedema 27 (ULL-27) is a patient-reported questionnaire that evaluates the QoL of patients with upper limb lymphedema in three domains (physical, psychological, and social). ULL-27 was rated “sufficient” for content validity, structural validity, and internal consistency. However, it was rated “indeterminate” for reliability and “insufficient” for construct validity. The “indeterminate” rating was given because they were not reporting the reliability to result in a preferred measure, such as intraclass correlation (ICC) or weighted Kappa (r = 0.40, *p* > 0.05). Meanwhile, the “insufficient” rating was given because less than 75% of the results were aligned with the hypotheses. ULL-27 quality of evidence was scored “low” for content validity and “very low” for structural validity and reliability. These scores were given due to the lack of information on the content validation process and the insufficient sample size of reliability (<50). Furthermore, there was only one “inadequate” quality study on structural validity and reliability [56,57].

Upper Limb Lymphedema Quality of Life Questionnaire (ULL-QoL) is a self-reported tool to measure the physical and emotional well-being of patients with upper limb lymphedema. It was rated “sufficient” for content validity, structural validity, internal consistency, reliability, construct validity, and responsiveness. However, the quality evidence of reliability and responsiveness were scored “very low” due to insufficient sample size (<50 for reliability and <100 for responsiveness). The score was given because there was only one study with “adequate” quality on reliability and only one methodologically “doubtful” study on responsiveness [58].

LYMPH-Q Upper Extremity is a patient-reported questionnaire that measures QoL among women with BCRL. LYMPH-Q consists of six independently functioning scales (appearance, function, psychological, symptoms, information, and arm sleeve), which means that only scales relevant to the patient’s situation need to be completed. Higher scales for LYMPH-Q scales indicated a better quality of life. It was rated “sufficient” for content validity, reliability, and construct validity. Meanwhile, the other measurement properties received various ratings: “insufficient” for structural validity, which was given because the study provided not enough information on the model fit; “indeterminate” for internal consistency, as the result of the structural validity “insufficient” rating. LYMPH-Q received “very low” for the quality of evidence of reliability because it has a low sample size (<100) and only one study with “doubtful” quality. Furthermore, similar to most of the PROMs reported in this review, LYMPH-Q was rated as “high” for its internal consistency and construct validity [59].

## 4. Discussion

Our review aims to assess the psychometric properties quality of QoL questionnaires and propose the most valid and reliable PROM for clinical and research use. To our knowledge, this is the first systematic review and critical appraisal of published studies reporting the psychometric properties of PROMs measuring BCRL patients’ QoL that utilized an updated COSMIN guideline and checklist.

Our findings indicated that most of the PROMs were evident in a few measurement properties only, such as content validity, structural validity, internal consistency, reliability, and hypothesis testing for construct validity. There was inadequate evidence on cross-cultural validity, measurement error, criterion validity, and responsiveness. A total of thirteen studies [41,42,43,44,45,46,47,52,53,55,56,57] evaluated the translated version of the PROMs, but cross-cultural validity has not yet been assessed. Cross-cultural validity should be assessed in these translation studies because it is essential to know whether the translated versions assess in the same manner as their original version. Measurement error needs to be evaluated to determine actual changes from systematic and random error so that the clinician can be more confident of the instrument’s reliability. Criterion validity is required because without it, a clinician could not be assured whether the instrument is already well-reflecting the gold standard. Responsiveness is important to be investigated to detect any change in the assessment following the interventions received by patients. The diverse quality of measurement properties in the included studies might be the result of a different approach used by the authors. This review revealed that only six studies use the COSMIN recommendations as their guideline in developing and validating the PROMs [48,49,50,51,52,53,58,59]. Other studies that translated and validated PROMs to other languages also used different translation guidelines [41,42,43,44,45,46,47,52,53,55,56,57].

According to Prinsen et al., recommendations on the most suitable PROM for use both in clinical and research settings can be formulated by categorizing the included PROMs into three categories: (A) PROMs that have the potential to be recommended as the most suitable PROM for the construct and population of interest (i.e., PROMs with evidence for sufficient content validity (any level) and at least low evidence for sufficient internal consistency); (B) PROMs that may have potential to be recommended, but further validation studies are needed (i.e., PROMs categorized not in A or C); (C) PROMs that should not be recommended (i.e., PROMs with high quality of evidence for insufficient measurement properties) [29,30]. Based on the quality assessments, we categorized the included PROMs into each category: (A) LLIS ver.1 [44,45], Lymph-ICF-UL [48,49,50,51,52,53], and ULL-QoL [58]; (B) LYMQOL-Arm [41,42], LLIS ver.2 [46,47]; (C) LSIDS-A [54,55] and ULL-27 [56,57]. They also advised recommending only one most suitable PROM. In case there are more than one PROMs that are difficult to differentiate in terms of quality, the one with the best evidence for content validity could be chosen as the most suitable instrument. It is also recommended that feasibility or interpretability aspects should be taken into consideration in the selection process [29,30].

Feasibility is the ease of administration of the PROM, given the time or money constraints. Feasibility aspects include: patient’s comprehensibility, clinician’s comprehensibility, type and ease of administration, length of the instrument, completion time, patient’s required mental and physical ability level, ease of standardization, ease of score calculation, copyright, cost of instrument, required equipment, availability in different settings, and regulatory agency’s requirement for approval. Interpretability is the degree to which one can assign qualitative meaning to a PROM’s quantitative scores or change in scores. Interpretability can be obtained from the following information: distribution of scores in the study population, percentage of missing items and percentage of missing total scores, floor and ceiling effects, scores and change scores available for relevant subgroups, minimal important change (MIC) or minimal important difference (MID), and information on response shift [30].

Among the three PROMs that we categorized as “A”, Lymph-ICF-UL [49,50,51,52,53,54] has the best evidence for content validity with “high” quality of evidence at any level (relevance, comprehensiveness, and comprehensibility). In terms of feasibility aspects, Lymph-ICF-UL has short, clear, and straightforward questions and an 11-point numerical scale that can be easily understood by the patients and the clinicians. The questionnaire also comes with an easy score calculation that is available in Excel formula. Lymph-ICF-UL only took 5–10 min to be completed and is available in various languages [48,49,50,51,52,53]. The other two PROMs are less suitable because: they only have “moderate” quality of evidence for the content validity; LLIS ver.1 [44,45] was validated in a population other than BCRL; ULL-QoL [58] has less-detailed daily activities-related questions (e.g., work activities, leisure activities) compared to Lymph-ICF-UL (i.e., clean, iron, work in the garden, perform computer work, drive a car, ride a bike), making it a little hard to address the patients’ difficulties in some daily activities. However, we are unable to compare the interpretability of the three PROMs due to the lack of information provided in the included studies. Overall, we consider Lymph-ICF-UL as the most suitable PROM to assess QoL in BCRL patients.

Based on the quality of evidence assessments, we found that Lymph-ICF-UL [48,49,50,51,52,53] had assessed seven of nine measurement properties suggested by COSMIN: content validity, structural validity, internal consistency, reliability, measurement error, hypothesis testing for construct validity, and responsiveness. Moreover, the overall rating of these measurement properties was mostly “sufficient” with “high” evidence levels. The structural validity was supported with exploratory factor analysis with acceptable factor loadings. The internal consistency of Lymph-ICF-UL was acceptable to excellent, with Cronbach’s alpha value ranging from 0.72 to 0.98. At the same time, the test-retest reliability was also considered good to very good with ICCs ranging from 0.79 to 0.95. Lymph-ICF-UL was also the only PROM reporting measurement error with the overall results of SEM = 4.51–12.6 and SDC = 12.5–34.91. The results for construct validity via hypothesis testing revealed that Lymph-ICF-UL has a moderate to high correlation with other PROMs measuring a similar construct. In terms of internal and external responsiveness, Lymph-ICF-UL was proven to be responsive to change after BCRL treatments.

Moreover, our result was in concordance with a systematic review [21] which indicated that lymphedema-specific questionnaires have strong psychometric properties and offer greater validity and reliability in measuring QoL of BCRL patients. A lymphedema-specific questionnaire contains items that address the patients’ complaints more precisely than the generic and cancer-specific questionnaire. The Lymph-ICF-UL domains (physical function, mental function, household, mobility, and social activities) are developed based on the International Classification of Functioning, Disability, and Health domains recommended by WHO [60].

Recommendation of PROM does not only depend on the measurement properties evaluation, but it also considers the other aspects (i.e., feasibility and interpretability aspects). Interpretability and feasibility are non-formal measurement properties because they do not refer to the quality of a PROM. Hence, they are only described and not evaluated. Both are important aspects that should be taken into account in selecting the most appropriate questionnaire, because: poor patient’s and clinician’s comprehensibility may indicate insufficient content validity; floor and ceiling effects can result in insufficient reliability.

This review’s strength is that compared to other reviews by Cornelissen et al., which only assess the completeness of the PROM by assessing the number of domains [32], this review provides a focused and comprehensive assessment of PROMs’ measurement properties as recommended by COSMIN [29]. A susceptible search strategy developed by Terwee et al. [34] was applied to identify relevant studies. In addition, this is the first study to focus on the breast cancer-related lymphedema population solely.

However, our decision not to consider certain lymphedema severity as the inclusion criteria might be the limitation of this review. This limitation could make the result difficult to generalize to all stages of severity. Our rationale is that most studies did not specify the severity of their study population, making it difficult for us to identify it. Another limitation is the possibility of publication bias due to the assumption that if the PROMs validation studies were not identified through our search, these had not been carried out. Furthermore, since this study focuses only on PROMs assessing QoL in the BCRL population, other PROMs measuring QoL might be omitted if they were not explicitly assessed in the BCRL population.

## 5. Conclusions

This systematic review provides an overview of the psychometric properties of updated PROMs assessing QoL in BCRL populations. Lymph-ICF-UL was found to have assessed most of the measurement properties as suggested by COSMIN and showed a “sufficient” overall rating with a high-quality level of evidence. Thus, we consider Lymph-ICF-UL to be a suitable PROM in measuring the QoL of patients with BCRL in either clinical or research settings.

## Figures and Tables

**Table 1:** COSMIN definitions of measurement properties.

Measurement Properties	Definition *
Content validity	The degree to which the content of a PROM is an adequate reflection of the construct to be measured
Structural validity	The degree to which the scores of a PROM are an adequate reflection of the dimensionality of the construct to be measured
Internal consistency	The degree of the interrelatedness among the items
Cross-cultural validity	The degree to which the performance of the items on a translated or culturally adapted PROM is an adequate reflection of the original version of the PROM
Reliability	The proportion of the total variance in the measurements which is due to “true” differences between patients
Measurement error	The systematic and random error of a patient’s score that is not attributed to true changes in the construct to be measured
Criterion validity	The degree to which the scores of a PROM are an adequate reflection of a “gold standard”
Hypothesis testing for construct validity	The degree to which the scores of a PROM are consistent with the hypothesis (for instance with regard to internal relationships, relationships to scores of other instruments, or differences between relevant groups) based on the assumption that the PROM validly measures the construct to be measured
Responsiveness	The degree to which the scores of a PROM to detect change over time in the construct is to be measured

* Definitions were adapted from COSMIN manual for systematic reviews of PROMs [30]; PROMs = patient-reported outcome measures.

**Table 2:** Criteria for good measurement properties.

Measurement Properties	Rating	Criteria *
Structural validity	+	**CTT:** CFA: CFI or TLI or comparable measure >0.95 OR RMSEA <0.06 OR SRMR <0.08^2^ **IRT/Rasch:** No violation of unidimensionality: CFI or TLI or comparable measure >0.95 OR RMSEA <0.06 OR SRMR <0.08^2^ *AND* no violation of monotonicity: adequate looking graphs OR item scalability >0.30 *AND* adequate model fit: **IRT:** χ^2^ > 0.01 **Rasch:** infit and outfit mean squares ≥0.5 and ≤1.5 OR Z-standardized values >−2 and <2
	?	**CTT:** Not all information for ‘+’ reported **IRT/Rasch:** Model fit not reported
	−	Criteria for ‘+’ not met
Internal consistency	+	At least low evidence for sufficient structural validity AND Cronbach’s alpha(s) ≥0.70 for each unidimensional scale or subscale
	?	Criteria for “At least low evidence for sufficient structural validity” not met
	−	At least low evidence for sufficient structural validity AND Cronbach’s alpha(s) < 0.70 for each unidimensional scale or subscale
Reliability	+	ICC or weighted Kappa ≥ 0.70
	?	ICC or weighted Kappa not reported
	−	ICC or weighted Kappa < 0.70
Measurement error	+	SDC or LoA < MIC
	?	MIC not defined
	−	SDC or LoA > MIC
Hypothesis testing for construct validity	+	The result is in accordance with the hypothesis
	?	No hypothesis defined (by the review team)
	−	The result is not in accordance with the hypothesis
Cross-cultural validity	+	No important differences found between group factors (such as age, gender, language) in multiple group factor analysis OR no important DIF for group factors (McFadden’s R^2^ < 0.02)
	?	No multiple group factor analysis OR DIF analysis performed
	−	Important differences between group factors were found
Criterion validity	+	Correlation with gold standard ≥ 0.70 OR AUC ≥ 0.70
	?	Not all information for ‘+’ reported
	−	Correlation with gold standard < 0.70 OR AUC < 0.70
Responsiveness	+	The result is in accordance with the hypothesis OR AUC ≥ 0.70
	?	No hypothesis defined (by the review team)
	−	The result is not in accordance with the hypothesis OR AUC < 0.70

* Criteria adapted from COSMIN manual for systematic reviews of PROMs [30]; “+” = sufficient, “−” = insufficient, “?” = indeterminate, AUC = area under the curve, CFA = confirmatory factor analysis, CFI = comparative fit index, CTT = classical test theory, DIF = differential item functioning, ICC = intraclass correlation coefficient, IRT = item response theory, LoA = limits of agreement, MIC = minimal important change, RMSEA: root mean square error of approximation, SEM = standard error of measurement, SDC = smallest detectable change, SRMR: standardized root mean residuals, TLI = Tucker–Lewis index.

**Table 3:** Characteristics of included studies.

Author (ref)	Country	PROM	Objective of Study	Sample Size	Age Mean ± SD (Range) Year	Gender (% Female)	Lymphedema Characteristics		
							Type	Duration	Severity
Bakar et al. 2017 [41]	Turkey	LYMQoL-Arm A	To translate the English version of LYMQoL to Turkish and to test the reliability and validity of the Turkish version of LYMQoL among patients with BCRL in Turkey	4 translators 20 patients for pilot study 65 patients for validation studies	50.6 ± 12.45 (24–75)	100%	BCRL	4.32 ± 3.06 (1–18) years	Not specified
Karayurt et al. 2021 [42]	Turkey	LYMQoL-Arm A	To adapt Quality of Life Measure for Limb Lymphedema-Arm (LYMQoL-Arm) into Turkish (TR) and test its validity and reliability	6 translators 5 experts for content validity 10 patients for pilot study 109 patients for structural validity, construct validity, internal consistency, and reliability analysis	55.69 ± 9.33 (35–79)	100%	BCRL	3.28 ± 2.91 (1–13) years	Mild-severe
Borman et al. 2018 [43]	Turkey	LYMQoL-Arm B	To translate and validate the LYMQoL-Arm for Turkish breast cancer patients with lymphedema	4 experts for the translation process 30 patients for pre-testing 135 patients for validation studies	51.8 ± 9.8 (31–82)	100%	BCRL	21.1 ± 38.7 (0.2–164) months	Stage 1–3
Degirmenci et al. 2019 [44]	Turkey	LLIS ver 1	To investigate the validity and reliability of the Turkish adaptation of the LLIS in patients with lymphedema	2 translators 10 patients for cognitive debriefing Patients for validation studies → UL = 79; LL = 27	53.6 ± 11.8 (28–83)	97.5% for UL group 96.3% for LL group	70.7% BCRL; 0.94% lymphoma; 25.4% LL lymphedema	Median = 24 (1–396) months for UL Median = 54 (1–384) months for LL	Stage 1–2 for UL Stage 1–3 for LL
Haghighat et al. 2018 [45]	Iran	LLIS ver 1	To validate the Persian version of the LLIS questionnaire	2 translators 10 patients for face validity 9 experts for content validity 203 for construct validity and internal consistency 13 for test-retest reliability 200 LE and 200 non-LE for discriminant validity 46 (LLIS vs. EORTC-QLQ-C30) and 400 (LLIS vs. SF-36) for convergent validity	53.28 ± 10.95	100%	Unilateral BCRL	Not specified	Not specified
Orhan et al. 2019 [46]	Turkey	LLIS ver 2	To translate and culturally adapt the LLIS ver 2 into Turkish and perform a psychometric evaluation of the Turkish LLIS ver 2 in patients with BCRL	10 experts for the translation process 20 patients for pilot testing 78 patients with LE 35 patients without LE for validation studies	56.5 ± 10.21	100%	69.02% BCRL; 30.9% non-LE	0–6 mo: 20.5% 6–12 mo: 21.8% 1–3 yr: 24.4% 3–5 yr: 19.2% 5–10 yr: 11.5% >10 yr: 2.6%	Not specified
Sharour 2020 [47]	Jordan	LLIS ver 2	To translate and validate an Arabic version of the LLIS	3 experts for the translation process 90 patients for validation studies	44.1 ± 1.10	100%	BCRL	0–6 mo: 80% 6–12 mo: 17.8% 1–2 yr: 2.2%	Not specified
Devoogdt et al. 2011 [48]	Belgium	Lymph-ICF-UL	To investigate the reliability (test-retest, internal consistency, measurement variability) and validity (content and construct) of the newly developed Lymph-ICF in breast cancer patients with lymphedema	20 patients for phase 1 (generating items) 29 patients for phase 2 (validation of the pilot version) 3 translators for phase 3 (translation from Dutch to English) 60 patients LE and 30 patients non-LE for validation studies	61.2 ± 10.0 (objective LE); 56.7 ± 9.3 (subjective LE); 58.3 ± 11.9 (non-LE)	100%	66% BCRL; 33.3% non-LE	Objective LE = 41 ± 64 months Subjective LE = 19 ± 34 months	Not specified
Grarup et al. 2018 [49]	Denmark	Lymph-ICF-UL	To translate and culturally adapt the original Dutch version of Lymph-ICF into Danish and examine its content validity and reliability	4 experts for the translation process 10 patients for cognitive debriefing 52 patients for validation studies	61 ± 12.4 (validation studies); 61.5 ± 9.7 (cognitive debriefing)	100%	BCRL	15.5 ± 58 months for validation studies 24 ± 31 months for cognitive interview	Mild to severe
de Vrieze et al. 2019 [50]	Belgium	Lymph-ICF-UL	To examine the validity and reliability of the Lymph-ICF-UL with NRS in patients with BCRL	56 patients	62 ± 10	100%	BCRL	34.5 months	Stage I, IIa, IIb
de Vrieze et al. 2020 [51]	Belgium	Lymph-ICF-UL	To examine the internal and external responsiveness of the Lymph-ICF-UL in patients with BCRL	95 patients	62 ± 10	100%	BCRL	53 ± 42.5	Stage I, IIa, IIb
de Vrieze et al. 2021 [52]	Belgium	Lymph-ICF-UL	To perform a cross-cultural validation of the Lymph-ICF-UL French version in patients with BCRL of the arm and/or hand	3 experts and 3 patients for the translation process 50 patients for validation studies	64 ± 11	100%	BCRL	78 months	Stage I, IIa, IIb
Zhao et al. 2022 [53]	China	Lymph-ICF-UL	To translate the Lymph-ICF-UL into a Chinese version and subsequently test its reliability and validity among patients with BCRL in a Chinese context	5 translators 15 patients for pilot testing 6 experts for content validity 155 patients LE and 90 patients non-LE for validation studies	26–70	100%	63.2% BCRL; 36.7% non-LE	2–19 months	Stage 0–3
Ridner and Dietrich 2015 [54]	USA	LSIDS-A	To develop and examine the psychometric properties (validity and reliability) of LSIDS-A in breast cancer patients experiencing upper limb lymphedema	128 for preliminary testing 236 for validation studies	58.9 ± 11.0	100%	BCRL	Not specified	84.5% had stage II lymphedema
Deveci et al. 2021 [55]	Turkey	LSIDS-A	To adapt LSIDS-A into Turkish and to test its validity and reliability in patients with BCRL	6 translators 5 experts for content validity 20 patients for pilot testing 186 patients for structural validity, construct validity, and internal consistency	55.4 ± 10.2 (20–80)	100%	BCRL	48.8 ± 49.5 (1–204) months	Not specified
Viehoff and Wittink 2008 [56]	Netherland	ULL-27	To translate the ULL27 into Dutch and to assess its internal consistency and validity for Dutch patients with upper limb lymphedema	3 translators 5 patients for cognitive interview 84 patients LE and 61 patients non-LE for validation studies	59 ± 11.79 (34–80)	100%	BCRL	35.51 ± 45.14 (0.5–276) months	Not specified
Vatansever et al. 2020 [57]	Turkey	ULL-27	To perform translation, cultural adaptation, and validation of ULL-27 in Turkish-speaking population of BCRL; To assess QoL of Turkish BCRL patients	4 translators 15 patients for cognitive interview 81 patients for validation studies	54.96 ± 11.35	100%	BCRL	23.12 ± 30.88 months	Mild to severe
Williams et al. 2018 [58]	Australia	ULL-QoL	To develop PROM specific to the assessment of HRQoL associated with upper limb lymphedema and assess its psychometric properties	24 patients for PROM development 5 patients and 16 therapists for content validity 103 patients for reliability, construct validity, and responsiveness	60.3 ± 13.0 (23–86)	97%	99% BCRL, 1% Non-Hodgkin’s lymphoma	Not specified	Not specified
Klassen et al. 2021 [59]	Canada	LYMPH-Q Upper Extremity	To describe the development and psychometric validation of the LYMPH-Q Upper Extremity Module	15 patients for qualitative interviews 16 patients for content validity 3222 patients for structural validity, construct validity, internal consistency, and reliability	40–70	100%	BCRL	≤4 yrs: 31% 5–9 yrs: 36.7% ≥10 yrs: 32.3%	Mild to severe

SD = standard deviation, LYMQoL-Arm = Lymphedema Quality of Life Tool-Arm, BCRL = breast cancer-related lymphedema; LLIS 1 = Lymphedema Life Impact Scale version 1, UL = upper limb, LL = lower limb, LE = lymphedema, EORTC QLQ-C30 = European Organization for Research and Treatment of Cancer Quality of Life Questionnaire Core 30, SF-36 = 36-items Short Form Health Survey, LLIS 2 = Lymphedema Life Impact Scale version 2, CVI = chronic venous insufficiency, DVT = deep vein thrombosis, Lymph-ICF-UL = Lymphedema Functioning, Disability, and Health Questionnaire for Upper Limb, Ly-QLI = Lymphedema Quality of Life Inventory, LSIDS-A = Lymphedema Symptom Intensity and Distress Survey-Arm, PROM = patient-reported outcome measure.

**Table 4:** Characteristics of included PROMs.

PROM	Ref	Country (Language in which the PROM was Evaluated)	No of Items	Subscales	Recall Period	Response Option	Scoring	Original Language	Available Translation
LYMQOL-Arm A (Lymphedema Quality of Life Tool-Arm A)	Bakar et al. 2017 [41]	Turkey	21	4 domains: function, appearance, symptoms, mood	Not specified	Domains: 4-point Likert scale (1–4); overall QoL: 0–10 scale	Total score of all domains and overall QoL score	English	Turkish
	Karayurt et al. 2021 [42]	Turkey	21	4 subscales: symptom, body image/appearance, function, mood	Not specified	Domains: 4-point Likert scale (1–4); overall QoL: 0–10 scale	Total score of all domains and overall QoL score	English	Turkish
LYMQoL-Arm B (Lymphedema Quality of Life Tool-Arm B)	Borman et al. 2018 [43]	Turkey	28 (adding 7 sub-questions)	4 domains: function, appearance, symptoms, mood	Not specified	Domains: 4-point Likert scale (1–4); overall QoL: 0–10 scale	Total score of all domains and overall QoL score	English	Turkish
LLIS 1 (Lymphedema Life Impact Scale version 1)	Degirmenci et al. 2019 [44]	Turkey	18	3 subscales: physical, psychosocial, functional	Not specified	5-point Likert scale (1–5)	Total score, subscale score	English	Turkish, Persian
	Haghighat et al. 2018 [45]	Iran	18	3 subscales: physical, psychosocial, functional	Not specified	5-point Likert scale (1–5)	Total score, subscale score	English	Turkish, Persian
LLIS 2 (Lymphedema Life Impact Scale version 2)	Orhan et al. 2019 [46]	Turkey	18	3 subscales: physical, psychosocial, functional	Not specified	5-point Likert scale (0–4)	Total score, subscale score	English	Turkish, Arabic
	Sharour 2020 [47]	Jordan	18	3 subscales: physical, psychosocial, functional	Not specified	5-point Likert scale (0–4)	Total score, subscale score	English	Turkish, Arabic
Lymph-ICF-UL (Lymphedema Functioning, Disability, and Health Questionnaire for Upper Limb)	Devoogdt et al. 2011 [48]	Belgium	29	5 domains: physical, mental, household, mobility, life, and social activities	Complaints during the last 2 weeks	Visual Analog Scale (VAS) 0–100 mm	Total score, domain score	Dutch	English, Danish, French, Chinese
	Grarup et al. 2018 [49]	Denmark	29	5 domains: physical, mental, household, mobility, life, and social activities	Complaints during the last 2 weeks	Visual Analog Scale (VAS) 0–100 mm	Total score, domain score	Dutch	English, Danish, French, Chinese
	de Vrieze et al. 2019 [50]	Belgium	29	5 domains: physical, mental, household, mobility, life, and social activities	Complaints during the last 2 weeks	11-point Likert scale (0–10)	Total score, domain score	Dutch	English, Danish, French, Chinese
	de Vrieze et al. 2020 [51]	Belgium	29	5 domains: physical, mental, household, mobility, life, and social activities	Complaints during the last 2 weeks	11-point Likert scale (0–10)	Total score, domain score	Dutch	English, Danish, French, Chinese
	de Vrieze et al. 2021 [52]	Belgium	29	5 domains: physical, mental, household, mobility, life, and social activities	Complaints during the last 2 weeks	11-point Likert scale (0–10)	Total score, domain score	Dutch	English, Danish, French, Chinese
	Zhao et al. 2022 [53]	China	29	5 domains: physical, mental, household, mobility, life, and social activities	Complaints during the last 2 weeks	11-point Likert scale (0–10)	Total score, domain score	Dutch	English, Danish, French, Chinese
LSIDS-A (Lymphedema Symptom Intensity and Distress Survey-Arm)	Ridner and Dietrich 2015 [54]	USA	36	7 clusters: soft tissue sensation, neurological sensation, function, biobehavioral, resource, sexuality, activity	Reflective period of 1 week	Yes/no response, if ‘yes’ then 1–10 rating was solicited	Overall score, cluster score, intensity, and distress score	English	Turkish
	Deveci et al. 2021 [55]	Turkey	36	7 clusters: soft tissue sensation, neurological sensation, function, biobehavioral, resource, sexuality, activity	Reflective period of 1 week	Yes/no response, if ‘yes’ then 1–10 rating was solicited	Overall score, cluster score, intensity, and distress score	English	Turkish
ULL27 (Upper Limb Lymphedema 27)	Viehoff and Wittink 2008 [56]	Netherlands	27	3 domains: physical, psychological, social	Not specified	5-point Likert scale	Total score, domain score	French	Dutch, Turkish, English
	Vatansever et al. 2020 [57]	Turkey	27	3 domains: physical, psychological, social	Not specified	5-point Likert scale	Total score, domain score	French	Dutch, Turkish, English
ULL-QoL (Upper Limb Lymphedema Quality of Life Questionnaire)	Williams et al. 2018 [58]	Australia	14	2 dimensions: physical well-being, emotional well-being	Over the previous 2 weeks	5-point Likert scale	Total score, dimension score	English	None
LYMPH-Q Upper Extremity	Klassen et al. 2021 [59]	Canada	68	6 scales: - appearance - function - psychological - symptoms - information - arm sleeve	Now (appearance); past week (function, psychological, symptoms); N/A (information); most recent (arm sleeve)	4 response options for each scale: - extremely, moderately, a little, not at all (appearance and function) - always, often, sometimes, never (psychological) - severe, moderate, mild, none (symptoms) - very dissatisfied, somewhat dissatisfied, somewhat satisfied, very satisfied (information and arm sleeve)	Scale score	English	None

PROM = patient-reported outcome measure, QoL = quality of life.

**Table 5:** (**a**) COSMIN ratings on methodology quality and results per measurement property. (**b**) COSMIN ratings on methodology quality and results per measurement property (continued). (**c**) COSMIN ratings on methodology quality and results per measurement property (continued).

(a)									
COSMIN Measurement Properties	LYMQoL-Arm A [41,42]			LYMQoL-Arm B [43]			LLIS ver 1 [44,45]		
	Studies (Meth Qual Rating)	Results (Rating)	Summary of Results (Overall Rating)	Studies (Meth Qual Rating)	Results (Rating)	Summary of Results (Overall Rating)	Studies (Meth Qual Rating)	Results (Rating)	Summary of Results (Overall Rating)
	V/A/D/I *	+/−/? **	+/−/±/? **	V/A/D/I *	+/−/? **	+/−/±/? **	V/A/D/I *	+/−/? **	+/−/±/? **
Content validity	Bakar 2017 (D)	Relevance: (+) Comprehensiveness: (+) Comprehensibility: (+)	Content validity: (+)	Borman 2018 (D)	Relevance: (+) Comprehensiveness: (+) Comprehensibility: (+)	Content validity: (+)	Degirmenci 2019 (D)	Relevance: (+) Comprehensiveness: (+) Comprehensibility: (+)	Content validity: (+)
	Karayurt 2021 (D)	Relevance: (+) Comprehensiveness: (+) Comprehensibility: (+)					Haghighat 2018 (D)	Relevance: (+) Comprehensiveness: (+) Comprehensibility: (+)	
Structural validity	Bakar 2017 (I)	EFA → factor 1 = 0.624–0.912; factor 2 = 0.587–0.876; factor 3 = 0.376–0.866; factor 4 = 0.788–0.861 (+)	4 factors with acceptable factor loadings (+)	Borman 2018 (I)	CFA → CMIN/df: 1.733, RMSEA: 0.074, GFI: 0.782, IFI: 0.904, CFI: 0.902, TLI: 0.888 (−)	Criteria for model fit were not met (−)	Degirmenci 2019 (I)	EFA → factor 1 = 0.214–0.770; factor 2 = 0.571–0.818; factor 3 = 0.309–0.748 (+)	3 factors with acceptable factor loadings (+)
	Karayurt 2021 (A)	CFA → CMIN/df: 1.86, RMSEA: 0.089, SRMR: 0.09, CFI: 0.81, GFI: 0.74, AGFI: 0.68 (−)					Haghighat 2018 (V)	CFA → NFI: 0.856, NNFI: 0.894, CFI: 0.908, MFI: 0.909, RMSEA: 0.087; EFA → factor 1 = 0.621–0.884; factor 2 = 0.651–0.821; factor 3 = 0.443–0.631 (+)	
Internal consistency	Bakar 2017 (V)	Cronbach’s α (total) = 0.91; Cronbach’s α (domains) = 0.70–0.94 (+)	Cronbach’s α = 0.70–0.94 (+)	Borman 2018 (V)	Cronbach’s α = 0.85–0.90 (?)	(?)	Degirmenci 2019 (V)	Cronbach’s α (subscales) = 0.771–0.865; Cronbach’s α (total) = 0.916 (+)	Cronbach’s α = 0.771–0.879 (+)
	Karayurt 2021 (V)	Cronbach’s α (total) = 0.90; Cronbach’s α (domains) = 0.78–0.86 (+)					Haghighat 2018 (V)	Cronbach’s α = 0.853–0.879 (+)	
Cross-cultural validity/measurement invariance	N/A	N/A	N/A	N/A	N/A	N/A	N/A	N/A	N/A
Reliability	Bakar 2017 (A)	Test-retest: ICC (total) = 0.99; ICC (domains) = 0.98–0.99 (+)	Test-retest ICC = 0.92–0.99 (+)	Borman 2018 (V)	Test-retest: ICC (total) = 0.627; ICC (domains) = 0.451–0.714 (−)	(−)	Degirmenci 2019 (V)	Test-retest: ICC (subscales) = 0.963–0.985; ICC (total) = 0.991 (+)	Test-retest ICC = 0.855–0.991 (+)
							Haghighat 2018 (A)	Test retest: ICC (subscales) = 0.855–0.977; ICC (total) = 0.962 (+)	
Measurement error	N/A	N/A	N/A	N/A	N/A	N/A	N/A	N/A	N/A
Criterion validity	N/A	N/A	N/A	N/A	N/A	N/A	N/A	N/A	N/A
Hypothesis testing (for construct validity)	Bakar 2017 (V)	LYMQoL-Arm A and NHP r = 0.539–0.643, *p* < 0.05; LYMQoL-Arm A and Overall QoL r = −0.535 to −0.707, *p* < 0.05 (2+)	Result in line with 6 hypotheses, but not with 1 hypothesis (+)	Borman 2018 (V)	Convergent validity → LYMQoL-Arm B and EORTC-BR23 (body image, future, systemic complications, breast symptoms, arm symptoms) r = 0.203 to 0.637, *p* < 0.05; LYMQoL-Arm B and FACT-B4 r = −0.100 to −0.530, *p* < 0.05; Divergent validity → LYMQoL-Arm B and EORTC-BR23 (sexuality, hair loss) r = −0.017 to 0.214, *p* < 0.05 (3+)	Result in line with 3 hypotheses (+)	Degirmenci 2019 (V)	LLIS 1 and SF-12 r_s_ = −0.453 to −0.703, *p* < 0.01; LLIS 1 and EORTC QLQ-C30 r_s_ = 0.496–0.723, *p* < 0.01; LLIS 1 and DASH r_s_ = 0.580–0.785, *p* < 0.01 (3+)	Result in line with 5 hypotheses, but not with 1 hypothesis (+)
	Karayurt 2021 (V)	Known groups validity → the mean scores of LYMQoL-Arm A total (t = −4.628, *p* = 0.001), subscales symptom (t = −2.113, *p* = 0.038), body image/appearance (t = −5.247, *p* = 0.001), and function (t = −5.874, *p* = 0.001) in patients with severe LE were significantly higher than patients with mild LE, but no significant different in both groups’ mean scores for subscale mood (t = −0.776, *p* = 0.446) (4+, 1-)					Haghighat 2018 (V)	Discriminant validity → patients with LE showed higher impairments in all three subscales compared to those without LE, *p* < 0.01 for physical and functional subscales; Convergent validity → LLIS 1 and SF-36 r_s_ = −0.344 to −0.497, *p* < 0.01; LLIS 1 and EORTC QLQ-C30 r_s_ ≤ −0.388 to −0.723, *p* < 0.01 (2+, 1-)	
Responsiveness	N/A	N/A	N/A	N/A	N/A	N/A	N/A	N/A	N/A
(**b**)									
**COSMIN Measurement Properties**	**LLIS ver 2** [46,47]			**Lymph-ICF-UL** [48,49,50,51,52,53]			**LSIDS-A** [54,55]		
	**Studies (Meth Qual Rating)**	**Results (Rating)**	**Summary of Results (Overall Rating)**	**Studies (Meth Qual Rating)**	**Results (Rating)**	**Summary of Results (Overall Rating)**	**Studies (Meth Qual Rating)**	**Results (Rating)**	**Summary of Results (Overall Rating)**
	V/A/D/I *	+/−/? **	+/−/±/? **	V/A/D/I *	+/−/? **	+/−/±/? **	V/A/D/I *	+/−/? **	+/−/±/? **
Content validity	Orhan 2019 (D)	Relevance: (+) Comprehensiveness: (+) Comprehensibility: (+)	Content validity: (+)	Devoogdt 2011 (D)	Relevance: (+) Comprehensiveness: (+) Comprehensibility: (+)	Content validity: (+)	Ridner 2015 (D)	Relevance: (+) Comprehensiveness: (+) Comprehensibility: (+)	Content validity: (+)
	Sharour 2020 (D)	Relevance: (+) Comprehensiveness: (+) Comprehensibility: (+)		Grarup 2018 (A)	Relevance: (+) Comprehensiveness: (+) Comprehensibility: (+)		Deveci 2021 (A)	Relevance: (+) Comprehensiveness: (+) Comprehensibility: (+)	
				De Vrieze 2019 (D)	Relevance: (+) Comprehensiveness: (+) Comprehensibility: (+)				
				De Vrieze 2021 (D)	Relevance: (+) Comprehensiveness: (+) Comprehensibility: (+)				
				Zhao 2022 (A)	Relevance: (+) Comprehensiveness: (+) Comprehensibility: (+)				
Structural validity	Orhan 2019 (A)	EFA → factor 1 = 0.502–0.751; factor 2 = 0.401–0.787; factor 3 = 0.426–0.844 (+)	3 factors with acceptable factor loadings (+)	Zhao 2022 (A)	EFA → factor 1 = 0.648–0.784; factor 2 = 0.754–0.798; factor 3 = 0.419–0.802; factor 4 = 0.808–0.881; factor 5 = 0.457–0.739 (+)	5 factors with acceptable factor loadings (+)	Deveci 2021 (A)	CFA → for intensity scale: CMIN/df: 1.52, RMSEA: 0.056, SRMR: 0.19, CFI: 0.91, GFI: 0.83, IFI: 0.91, TLI: 0.90; for distress scale: CMIN/df: 1.55, RMSEA: 0.055, SRMR: 0.27, CFI: 0.90, GFI: 0.84, IFI: 0.90, TLI: 0.893 (+)	Model fit was acceptable (+)
	Sharour 2020 (D)	EFA → factor 1 = 0.65–0.76; factor 2 = 0.61–0.88; factor 3 = 0.60–0.72 (+)							
Internal consistency	Orhan 2019 (V)	Cronbach’s α (subscales) = 0.76–0.78; Cronbach’s α (total) = 0.89 (+)	Cronbach’s α = 0.76–0.923 (+)	Devoogdt 2011 (V)	Cronbach’s α (domains) = 0.72–0.92; Cronbach’s α (total) = 0.92 (+)	Cronbach’s α = 0.72–0.98 (+)	Ridner 2015 (V)	KR-20 (symptoms occurrence) = 0.88; Cronbach’s α (intensity score) = 0.93; Cronbach’s α (distress score) = 0.94 (+)	KR-20 = 0.83–0.88; Cronbach’s α = 0.68–0.94 (+)
	Sharour 2020 (V)	Cronbach’s α (subscales) = 0.861–0.901; Cronbach’s α (total) = 0.923 (+)		Grarup 2018 (V)	Cronbach’s α (domains) = 0.92–0.97; Cronbach’s α (total) = 0.98 (+)		Deveci 2021 (V)	KR-20 (symptoms occurrence) = 0.83; Cronbach’s α (intensity score) = 0.76–0.86; Cronbach’s α (distress score) = 0.68–0.86 (+)	
				De Vrieze 2019 (V)	Cronbach’s α (domains) = 0.89–0.98; Cronbach’s α (total) = 0.98 (+)				
				De Vrieze 2021 (V)	Cronbach’s α (domains) = 0.77–0.89; Cronbach’s α (total) = 0.95 (+)				
				Zhao 2022 (V)	Cronbach’s α (domains) = 0.789–0.910; Cronbach’s α (total) = 0.918 (+)				
Cross-cultural validity/measurement invariance	N/A	N/A	N/A	N/A	N/A	N/A	N/A	N/A	N/A
Reliability	Orhan 2019 (A)	Test-retest: ICC (subscales) = 0.88–0.93; ICC (total) = 0.91 (+)	Test-retest ICC = 0.88–0.93 (+)	Devoogdt 2011 (I)	Test-retest: ICC (domains) = 0.65–0.91; ICC (total) = 0.93 (+)	Test-retest ICC = 0.65–0.95 (+)	Ridner 2015 (D)	Test-retest: ICC (clusters) = 0.67–0.97; ICC (intensity) = 0.93; ICC (distress) = 0.92 (+)	Test-retest ICC = 0.67–0.93 (+)
				Grarup 2018 (D)	Test-retest: ICC (domains) = 0.88–0.94; ICC (total) = 0.95 (+)				
				De Vrieze 2019 (I)	Test-retest: ICC (domains) = 0.79–0.93; ICC (total) = 0.95 (+)				
				De Vrieze 2021 (I)	Test-retest: ICC (domains) = 0.66–0.95; ICC (total) = 0.91 (+)				
				Zhao 2022 (V)	Test-retest: ICC (domains) = 0.801–0.834; ICC (total) = 0.828 (+)				
Measurement error	N/A	N/A	N/A	Devoogdt 2011 (I)	Variability → SEM (total) = 4.8; SEM (domains) = 7.0–12.5; Clinically Important Changes → SDC (total) = 13.4; SDC (domains) = 19.4–34.6 (+)	SEM = 4.51–12.6; SDC = 12.5–34.91 (+)	N/A	N/A	N/A
				Grarup 2018 (D)	Variability → SEM (total) = 4.51; SEM (domains) = 5.69–10.21; Clinically Important Changes → SDC (total) = 12.5; SDC (domains) = 15.8–28.3 (+)				
				De Vrieze 2019 (I)	Variability → SEM (total) = 4.89; SEM (domains) = 6.31–12.31; Clinically Important Changes → SDC (total) = 13.56; SDC (domains) = 17.49–34.13 (+)				
				De Vrieze 2021 (I)	Variability → SEM (total) = 5.54; SEM (domains) = 6.28–12.6; Clinically Important Changes → SDC (total) = 15.35; SDC (domains) = 17.4–34.91 (+)				
Criterion validity	Orhan 2019 (V)	LLIS 2 (subscales) and LVD r = 0.30–0.36, *p* < 0.05; LLIS 2 (total) and LVD r = 0.39, *p* < 0.01 (−)	Weak correlation with gold measurement standard (LVD) r < 0.40 (−)	N/A	N/A	N/A	N/A	N/A	N/A
Hypothesis testing (for construct validity)	Orhan 2019 (V)	Convergent validity → LLIS 2 and LYMQOL (subscales) r = 0.52–0.82, *p* < 0.01; LLIS 2 and EORTC QLQ-C30 (functional and symptom) r = 0.67 to −0.85, *p* < 0.01; LLIS 2 and Quick-DASH r = 0.68–0.84, *p* < 0.01; Divergent validity → there was a significant difference in total score and all subscale scores between LE and non-LE groups, *p* < 0.05 (4+)	Result in line with 7 hypotheses (+)	Devoogdt 2011 (V)	Convergent validity → Lymph-ICF-UL and SF-36 (bodily pain, mental health, physical functioning, social functioning) r = −0.33 to −0.70; Divergent validity → Lymph-ICF-UL and SF-36 (role-emotional, mental health, physical functioning, role-physical) r = 0.03 to −0.42; Known-groups validity → the scores on 26 of 29 questions were significantly higher for LE patients compared to non-LE patients, *p* < 0.05 (40+, 5-)	Result in line with 75 hypotheses, but not with 15 hypotheses (+)	Ridner 2015 (V)	Convergent validity → LSIDS-A and FACT-G r_s_ = −0.20 to −0.53; LSIDS-A and FACT-B+4 r_s_ = −0.41 to −0.50; LSIDS-A and ULL-27 r_s_ = −0.29 to −0.52; LSIDS-A and FASQ r_s_ = 0.25–0.47; LSIDS-A and CES-D r_s_ = 0.29–0.65; LSIDS-A and FACT r_s_ = −0.46 to −0.50; LSIDS-A and POMS-SF r_s_ = 0.07–0.36; Divergent validity → LSIDS-A and MCSDS r_s_ = 0.01 to −0.25 (8+, 6-)	Result in line with 9 hypotheses, but not with 6 hypotheses (−)
	Sharour 2020 (V)	Convergent validity → LLIS 2 (total) and EORTC QLQ-C30 (functional and symptoms) r = 0.81 to −0.84; LLIS 2 (subscales) and EORTC QLQ-C30 (functional) r = −0.79 to −0.87; LLIS 2 (subscales) and EORTC QLQ-C30 (symptoms) r = 0.73–0.81 (3+)		De Vrieze 2019 (V)	Convergent validity → Lymph-ICF-UL and SF-36 (bodily pain, mental health, physical functioning, social functioning) r = −0.224 to −0.661; Divergent validity → Lymph-ICF-UL and SF-36 (role-emotional, mental health, physical functioning, role-physical) r_s_ = −0.191 to −0.607 (11+, 3-)		Deveci 2021 (V)	Known groups validity → there was a significantly higher mean score in patients with active LE compared to patients with latent LE (1+)	
				De Vrieze 2021 (V)	Convergent validity → Lymph-ICF-UL and SF-36 (bodily pain, mental health, physical functioning, social functioning) r_s_ = −0.156 to −0.704; Divergent validity → Lymph-ICF-UL and SF-36 (role-emotional, mental health, physical functioning, role-physical) r_s_ = −0.144 to −0.499 (9+, 5-)				
				Zhao 2022 (V)	Convergent validity → Lymph-ICF-UL and SF-36 (bodily pain, mental health, physical functioning, social functioning) r = −0.371 to −0.563; Lymph-ICF-UL and EORTC-QLQ-C30 r = 0.230 to −0.457; Divergent validity → Lymph-ICF-UL and SF-36 (role-emotional, mental health, physical functioning, role-physical) r = −0.102 to −0.376; Discriminant validity → patients with LE showed more impairments than patients without LE (*p* < 0.001) (15+, 2-)				
Responsiveness	N/A	N/A	N/A	De Vrieze 2020 (V)	Internal responsiveness → there were: a significant changes in mean total score between pre- and postintensive treatment (*p* < 0.05); no significant difference in mean total scores between pre- and posttreatment in stable group (*p* > 0.05); moderate responsiveness for total score (SRM = 0.65); External responsiveness → there were: a significant difference in mean change score between responders and non-responders after intensive treatment (*p* < 0.001); weak correlation between Δ-Lymph-ICF-UL and the GPE scores; MCID (total scores) = 9% (5+, 1-)	Results in line with 5 hypotheses, but not with 1 hypothesis (+)	N/A	N/A	N/A
(**c**)									
**COSMIN Measurement Properties**	**ULL27** [56,57]			**ULL-QoL** [58]			**LYMPH-Q Upper Extremity** [59]		
	**Studies (Meth Qual Rating)**	**Results (Rating)**	**Summary of Results (Overall Rating)**	**Studies (Meth Qual Rating)**	**Results (Rating)**	**Summary of Results (Overall Rating)**	**Studies (Meth Qual Rating)**	**Results (Rating)**	**Summary of Results (Overall Rating)**
	V/A/D/I *	+/−/? **	+/−/±/? **	V/A/D/I *	+/−/? **	+/−/±/? **	V/A/D/I *	+/−/? **	+/−/±/? **
Content validity	Viehoff 2008 (D)	Relevance: (+) Comprehensiveness: (+) Comprehensibility: (+)	Content validity: (+)	Williams 2018 (D)	Relevance: (+) Comprehensiveness: (+) Comprehensibility: (+)	Content validity: (+)	Klassen 2021 (D)	Relevance: (+) Comprehensiveness: (+) Comprehensibility: (+)	Content validity: (+)
	Vatansever 2020 (D)	Relevance: (+) Comprehensiveness: (+) Comprehensibility: (+)							
Structural validity	Vatansever 2020 (I)	CFA → RMSEA = 0.074; CFI = 0.97; IFI = 0.97; GFI = 0.96 (+)	Model fit was acceptable (+)	Williams 2018 (A)	EFA → factor 1 = 0.348–0.852; factor 2 = 0.375–0.870 (+)	2 factors with acceptable factor loadings (+)	Klassen 2021 (A)	Rasch: item fit was within ±2.5 for 27 of the 68 items (−)	Not all model fit was reported (−)
Internal consistency	Viehoff 2008 (V)	Cronbach’s α = 0.78–0.92 (?)	Cronbach’s α = 0.75–0.93 (+)	Williams 2018 (V)	Cronbach’s α = 0.87 (+)	(+)	Klassen 2021 (V)	Cronbach’s α (scales) = 0.89–0.97 (?)	(?)
	Vatansever 2020 (V)	Cronbach’s α (dimensions) = 0.75–0.90; Cronbach’s α (total) = 0.93 (+)							
Cross-cultural validity/measurement invariance	N/A	N/A	N/A	N/A	N/A	N/A	N/A	N/A	N/A
Reliability	Vatansever 2020 (I)	Test-retest r = 0.40, *p* > 0.05 (?)	r = 0.40, *p* > 0.05 (?)	Williams 2018 (A)	Test-retest ICC (total) = 0.93 (+)	(+)	Klassen 2021 (D)	Test-retest ICC (scales) = 0.92–0.96 (+)	(+)
Measurement error	N/A	N/A	N/A	N/A	N/A	N/A	N/A	N/A	N/A
Criterion validity	N/A	N/A	N/A	N/A	N/A	N/A	N/A	N/A	N/A
Hypothesis testing (for construct validity)	Viehoff 2008 (V)	Convergent validity → ULL-27 and RAND-36 r_s_ = 0.45–0.69; Discriminant validity → there was a significant difference in total scores and all domain scores between LE and non-LE groups, *p* < 0.001 (10+,1-)	Result in line with 11 hypotheses, but not with 14 hypotheses (−)	Williams 2018 (V)	Convergent validity → ULL-QoL and EQ-5D-3L r = −0.44 to −0.59; ULL-QoL (physical well-being) and SF-36 (PCS) r = −0.57; Divergent validity → ULL-QoL and % excess limb volume r = 0.12–0.18; ULL-QoL and SF-36 r = −0.31 to −0.43; ULL-QoL (emotional well-being) and EQ-5D-3L (utility scores) r = −0.50 (7+,1-)	Result in line with 7 hypotheses, but not with 1 hypothesis (+)	Klassen 2021 (V)	The correlation between symptoms, function, appearance, psychological, arm sleeve with each other was higher than with information (r = >0.50); All six scales were associated with increased severity of arm swelling, reporting of arm problem caused by cancer treatments, and wearing of a compression sleeve to reduce or prevent swelling in the past 12 months (3+, 1-)	Result in line with 3 hypotheses, but not with 1 hypothesis (+)
	Vatansever 2020 (V)	Convergent validity → ULL-27 and EORTC QLQ-C30 (QL2, PF2, RF2, EF, SF, FA, NV, PA, DY, SL, AP) r = −0.221 to −0.546, *p* < 0.001; ULL-27 and EORTC QLQ-BR23 (BRBI, BRFU, BRST) r = −0.248 (*p* < 0.005) to 0.348 (*p* < 0.001) (1+, 13-)							
Responsiveness	N/A	N/A	N/A	Williams 2018 (D)	LE transition to better → Mean change (SD of changes scores) = −5.4 (19.0) to −8.9 (17.7); MSRM = 0.30–0.64; LE transition to worse → Mean change (SD of changes scores) = 8.4 (13.8)–15.0 (27.7); MSRM = 0.61–0.83 (2+)	Result in line with 2 hypotheses (+)	N/A	N/A	N/A

* V = very good, A = adequate, D = doubtful, I = inadequate; ** + = sufficient, - = insufficient, **± =** inconsistent, ? = indeterminate; meth qual = methodological quality, LYMQOL-Arm A = Lymphedema Quality of Life Tool-Arm A, LYMQoL-Arm B = Lymphedema Quality of Life Tool-Arm B, LLIS 1 = Lymphedema Life Impact Scale version 1, LLIS 2 = Lymphedema Life Impact Scale version 2, Lymph-ICF-UL = Lymphedema Functioning, Disability, and Health Questionnaire for Upper Limb, LSIDS-A = Lymphedema Symptom Intensity and Distress Survey-Arm, ULL27 = Upper Limb Lymphedema 27, ULL-QoL = Upper Limb Lymphedema Quality of Life Questionnaire, EQ-5D-3L = EuroQol 5D three level version, EQ-VAS = EuroQol visual analogue scale, NHP = Nottingham health profile, EFA = exploratory factor analysis, ICC = intraclass correlation coefficient, CFA = confirmatory factor analysis, SF-36 = Short form 36, EORTC-BR23 = European Organization for Research and Treatment of Cancer Quality of Life Questionnaire Breast Cancer-Specific Version, FACT-B4 = Functional assessment of cancer therapy breast-4, CMIN/df = Satorra-Bentler scaled chi-square/degree of freedom, RMSEA = root mean square error of approximation, SRMR = standardized root mean square residual, GFI = goodness-of-fit index, IFI = incremental fit index, CFI = comparative fit index, TLI = Trucker-Lewis index, LEFS = Lower extremity functional scale, PCA = principal component analysis, UL = upper limb, LL = lower limb, EORTC QLQ-C30 = European Organization for Research and Treatment of Cancer Quality of Life Questionnaire Core 30, DASH = disabilities of arm shoulder and hand, NFI = Bentler-Bonnet normed fit index, NNFI = Bentler-Bonnet non-normed fit index, MFI = McDonald fit index, LVD = limb volume difference, SEM = standard error measurement, SRD = smallest real difference, KMO = Kaiser-Mayer Olkin, ADL = activity daily living, AUC = area under the ROC curve, CI = confidence interval, RP = rehabilitation program, LS = liposuction, FACT-G = Functional assessment of cancer therapy general, FASQ = Functional assessment screening questionnaire, CES-D = Center for epidemiologic studies-depression, POMS-SF = Profile of mood states short form, MCSDS = Marlowe–Crowne social desirability scale, KR-20 = Kuder–Richardson-20, SRM = standardized response mean, GPE = global perceived effect, MCID = minimal clinically important difference, MSRM = modified standardized response mean, N/A = not applicable.

**Table 6:** Quality of evidence for measurement properties of PROMs.

PROM * (ref)	Quality of Evidence Rating (GRADE **)										
	Content Validity			Structural Validity	Internal Consistency	Cross-Cultural Validity	Reliability	Measurement Error	Criterion Validity	Hypothesis Testing	Responsiveness
	**Relevance**	**Comprehensiveness**	**Comprehensibility**								
LYMQOL-Arm A [41,42]	Moderate	Moderate	Low	Moderate	High	N/A	Low	N/A	N/A	High	N/A
LYMQOL-Arm B [43]	Low	Low	Low	Very Low	High	N/A	Moderate	N/A	N/A	High	N/A
LLIS 1 [44,45]	Moderate	Moderate	Moderate	Moderate	Moderate	N/A	Moderate	N/A	N/A	Moderate	N/A
LLIS 2 [46,47]	Low	Low	Low	Low	Moderate	N/A	Very Low	N/A	Moderate	High	N/A
Lymph-ICF-UL [48,49,50,51,52,53]	High	High	High	Moderate	High	N/A	High	Low	N/A	High	Moderate
LSIDS-A [54,55]	Low	Moderate	Low	Moderate	High	N/A	Very Low	N/A	N/A	High	N/A
ULL-27 [56,57]	Low	Low	Low	Very Low	High	N/A	Very Low	N/A	N/A	High	N/A
ULL-QoL [58]	High	High	Moderate	Moderate	High	N/A	Very Low	N/A	N/A	High	Very Low
LYMPH-Q Upper Extremity [59]	Moderate	Moderate	Moderate	Moderate	High	N/A	Very Low	N/A	N/A	High	N/A

PROM * = patient-reported outcome measure; GRADE ** = Grading of Recommendation Assessment, Development, and Evaluation; LYMQOL-Arm A = Lymphedema Quality of Life Tool-Arm A, LYMQoL-Arm B = Lymphedema Quality of Life Tool-Arm B, LLIS 1 = Lymphedema Life Impact Scale version 1, LLIS 2 = Lymphedema Life Impact Scale version 2, Lymph-ICF-UL = Lymphedema Functioning, Disability, and Health Questionnaire for Upper Limb, LSIDS-A = Lymphedema Symptom Intensity and Distress Survey-Arm, ULL27 = Upper Limb Lymphedema 27, ULL-QoL = Upper Limb Lymphedema Quality of Life Questionnaire, N/A = not applicable.

## Data Availability

Data is provided within the article.

## Supplementary Materials

The details of database searches are available online at https://www.mdpi.com/article/10.3390/ijerph19052519/s1: Supplementary file S1:Database search (last updated 8 February 2022).
