# Supplementary material for: Psychometric Properties of Quality of Life Questionnaires for Patients with Breast Cancer-Related Lymphedema: A Systematic Review
Source: Int J Environ Res Public Health. 2022 Feb 22;19(5):2519. doi: 10.3390/ijerph19052519 (PMC8909332; doi:10.3390/ijerph19052519)
Supplement: Supplementary file 1 [file ijerph-19-02519-s001.zip › ijerph-1582804-supplementary.pdf]

Supplementary Files S1: Database search *(last updated February 8th, 2022)*

Ovid MEDLINE (239)
#1 (‘quality of life’)
(quality of life OR quality of living OR life quality OR welfare OR standard of living OR well-being)

#2 (‘breast cancer’)
(breast cancer OR mammary cancer OR breast carcinoma)

#3 (‘upper limb’)
(upper limb OR upper extremity OR arm OR hand)

#4 (‘lymphedema’)
(lymphedema OR lymphoedema OR lymphodema)

#5 (‘questionnaire’)
(questionnaire OR survey or inquiry OR question sheet OR enquiry)

#6 (‘measurement properties’)
(measurement properties) OR (accuracy) OR (accurate) OR (clinimetr*) OR (coefficient*) OR (consisten*) OR (correlated) OR (correlation*) OR (cronbach) OR (discrimina*) OR (interrater) OR (inter-rater) OR (intersession) OR (inter-session) OR (intertester) OR (inter-tester) OR (Intrarater) OR (intra-rater) OR (intratester) OR (intra-tester) OR (kappa) OR (Observer variation) OR (predictiv*) OR (propert*) OR (Psychometrics) OR (psychometr*) OR (reliab*) OR (repeatable) OR (repeatability) OR (Reproducibility of Results) OR (reproducible) OR (reproducibility) OR (responsive*) OR (Sensitivity and Specificity) OR (sensitive) OR (sensitivity) OR (spearman*) OR (specific) OR (specificity) OR (spearman) OR (subscale*) OR (suitable) OR (suitability) OR (test development) OR (test-retest) OR (useful*) OR (utility) OR (valid) OR (validity) OR (validat*) OR (Validation studies)

Combination search
#1 AND #2 AND #4 AND #5 AND #6 = #7

Limiters: ENGLISH LANGUAGE, FULL TEXT, HUMANS, FEMALES, ORIGINAL ARTICLES OR JOURNAL ARTICLE OR VALIDATION STUDY

EBSCOhost (259)
#1 (‘quality of life’)
SU ((quality of life OR quality of living OR life quality OR welfare OR standard of living OR well-being))

#2 (‘breast cancer’)
TX ((breast cancer OR mammary cancer OR breast carcinoma))

#3 (‘upper limb’)
TX ((questionnaire OR survey OR inquiry OR question sheet OR enquiry))

#4 (‘lymphedema’)
TX ((lymphedema OR lymphoedema OR lymphodema))

#5 (‘questionnaire’)
TX ((questionnaire OR survey or inquiry OR question sheet OR enquiry))

#6 (‘measurement properties’)
TX ((measurement properties) OR (accuracy) OR (accurate) OR (clinimetr*) OR (coefficient*) OR (consisten*) OR (correlated) OR (correlation*) OR (cronbach) OR (discrimina*) OR (interrater) OR (inter-rater) OR (intersession) OR (inter-session) OR (intertester) OR (inter-tester) OR (Intrarater) OR (intra-rater) OR (intratester) OR (intra-tester) OR (kappa) OR (Observer variation) OR (predictiv*) OR (propert*) OR (Psychometrics) OR (psychometr*) OR (reliab*) OR (repeatable) OR (repeatability) OR (Reproducibility of Results) OR (reproducible) OR (reproducibility) OR (responsive*) OR (Sensitivity and Specificity) OR (sensitive) OR (sensitivity) OR (spearman*) OR (specific) OR (specificity) OR (spearman) OR (subscale*) OR (suitable) OR (suitability) OR (test development) OR (test-retest) OR (useful*) OR (utility) OR (valid) OR (validity) OR (validat*) OR (Validation studies))

#7 (‘exclusion filter’)
TX ((biography) OR (case reports) OR (comment) OR (directory) OR (editorial) OR (interview) OR (lectures) OR (legal cases) OR (legislation) OR (letter) OR (news) OR (newspaper article) OR (patient education handout) OR (popular works) OR (congresses) OR (consensus development conference) OR (consensus development conference) OR (practice guideline))

Combination search
#1 AND #2 AND #4 AND #5 AND #6 NOT #7

Limiters: FULL TEXT, ENGLISH LANGUAGE, HUMANS, FEMALE, PUBLICATION TYPE JOURNAL ARTICLE OR VALIDATION STUDY

Scopus (109)
#1 (‘quality of life’)
(TITLE-ABS-KEY (“quality of life”) OR TITLE-ABS-KEY (“quality of living”) OR TITLE-ABS-KEY (“life quality”) OR TITLE-ABS-KEY (“welfare”) OR TITLE-ABS-KEY (“standard of living”) OR TITLE-ABS-KEY (“well-being”))

#2 (‘breast cancer’)
(TITLE-ABS-KEY (“breast cancer”) OR TITLE-ABS-KEY (“mammary cancer”) OR TITLE-ABS-KEY (“breast carcinoma”))

#3 (‘upper limb’)
(TITLE-ABS-KEY (“upper limb”) OR TITLE-ABS-KEY (“upper extremity”) OR TITLE-ABS-KEY (“arm”) OR TITLE-ABS-KEY (“hand”))

#4 (‘lymphedema’)
(TITLE-ABS-KEY (“lymphedema”) OR TITLE-ABS-KEY (“lymphoedema”) OR TITLE-ABS-KEY (“lymphodema”))

#5 (‘questionnaire’)

(TITLE-ABS-KEY (“questionnaire”) OR TITLE-ABS-KEY (“survey”) OR TITLE-ABS-KEY (“inquiry”) OR TITLE-ABS-KEY (“question sheet”) OR TITLE-ABS-KEY (“enquiry”))

#6 (‘measurement properties’)
(TITLE-ABS-KEY (“accuracy”) OR TITLE-ABS-KEY (“accurate”) OR TITLE-ABS-KEY (“clinimetr*”) OR TITLE-ABS-KEY (“coefficient*”) OR TITLE-ABS-KEY (“consisten*”) OR TITLE-ABS-KEY (“correlated”) OR TITLE-ABS-KEY (“correlation*”) OR TITLE-ABS-KEY (“cronbach”) OR TITLE-ABS-KEY (“discrimina*”) OR TITLE-ABS-KEY (“interrater”) OR TITLE-ABS-KEY (“inter-rater”) OR TITLE-ABS-KEY (“intersession”) OR TITLE-ABS-KEY (“inter-session”) OR TITLE-ABS-KEY (“intertester”) OR TITLE-ABS-KEY (“inter-tester”) OR TITLE-ABS-KEY (“Intrarater”) OR TITLE-ABS-KEY (“intra-rater”) OR TITLE-ABS-KEY (“intratester”) OR TITLE-ABS-KEY (“intra-tester”) OR TITLE-ABS-KEY (“kappa”) OR TITLE-ABS-KEY (“Observer variation”) OR TITLE-ABS-KEY (“predictiv*”) OR TITLE-ABS-KEY (“propert*”) OR TITLE-ABS-KEY (“Psychometrics”) OR TITLE-ABS-KEY (“psychometr*”) OR TITLE-ABS-KEY (“reliab*”) OR TITLE-ABS-KEY (“repeatable”) OR TITLE-ABS-KEY (“repeatability”) OR TITLE-ABS-KEY ("Reproducibility of Results") OR TITLE-ABS-KEY (“reproducible”) OR TITLE-ABS-KEY (“reproducibility”) OR TITLE-ABS-KEY (“responsive*”) OR TITLE-ABS-KEY ("Sensitivity and Specificity") OR TITLE-ABS-KEY (“sensitive”) OR TITLE-ABS-KEY (“sensitivity”) OR TITLE-ABS-KEY (“spearman*”) OR TITLE-ABS-KEY (“specific”) OR TITLE-ABS-KEY (“specificity”) OR TITLE-ABS-KEY (“spearman”) OR TITLE-ABS-KEY (“subscale*”) OR TITLE-ABS-KEY (“suitable”) OR TITLE-ABS-KEY (“suitability”) OR TITLE-ABS-KEY ("test development") OR TITLE-ABS-KEY (“test-retest”) OR TITLE-ABS-KEY (“useful*”) OR TITLE-ABS-KEY (“utility”) OR TITLE-ABS-KEY (“valid”) OR TITLE-ABS-KEY (“validity”) OR TITLE-ABS-KEY (“validat*”) OR TITLE-ABS-KEY (“Validation studies”))

#7 (‘exclusion filter’)
(TITLE-ABS-KEY (“biography”) OR TITLE-ABS-KEY (“case reports”) OR TITLE-ABS-KEY (“comment”) OR TITLE-ABS-KEY (“directory”) OR TITLE-ABS-KEY (“editorial”) OR TITLE-ABS-KEY (“interview”) OR TITLE-ABS-KEY (“lectures”) OR TITLE-ABS-KEY (“legal cases”) OR TITLE-ABS-KEY (“legislation”) OR TITLE-ABS-KEY (“letter”) OR TITLE-ABS-KEY (“news”) OR TITLE-ABS-KEY (“newspaper article”) OR TITLE-ABS-KEY (“patient education handout”) OR TITLE-ABS-KEY (“popular works”) OR TITLE-ABS-KEY (“congresses”) OR TITLE-ABS-KEY (“consensus development conference”) OR TITLE-ABS-KEY (“consensus development conference”) OR TITLE-ABS-KEY (“practice guideline”))

Combination search
#1 AND #2 AND #4 AND #5 AND #6 AND NOT #7

Limiters: HUMANS, ENGLISH, JOURNAL, ARTICLE

Web of Sciences (139)
#1 (‘quality of life’)
TS=(quality of life OR quality of living OR life quality OR welfare OR standard of living OR well-being)

#2 (‘breast cancer’)
TS=(breast cancer OR mammary cancer OR breast carcinoma)

#3 (‘upper limb’)
TS=(upper limb OR upper extremity OR arm OR hand)

#4 (‘lymphedema’)
TS=(lymphedema OR lymphoedema OR lymphodema)

#5 (‘questionnaire’)
TS=(questionnaire OR survey or inquiry OR question sheet OR enquiry)

#6 (‘measurement properties’)
TS=(measurement properties OR accuracy OR accurate OR clinimetr* OR coefficient* OR consisten* OR correlated OR correlation* OR cronbach OR discrimina* OR interrater OR inter-rater OR intersession OR inter-session OR intertester OR inter-tester OR Intrarater OR intra-rater OR intratester OR intra-tester OR kappa OR Observer variation OR predictiv* OR propert* OR Psychometrics OR psychometr* OR reliab* OR repeatable OR repeatability OR Reproducibility of Results OR reproducible OR reproducibility OR responsive* OR Sensitivity and Specificity OR sensitive OR sensitivity OR spearman* OR specific OR specificity OR spearman OR subscale* OR suitable OR suitability OR test development OR test-retest OR useful* OR utility OR valid OR validity OR validat* OR Validation studies)

#8 (‘exclusion filter’)
TS=(biography OR case reports OR comment OR directory OR editorial OR interview OR lectures OR legal cases OR legislation OR letter OR news OR newspaper article OR patient education handout OR popular works OR congresses OR consensus development conference OR consensus development conference OR practice guideline)

Combination search
#1 AND #2 AND #4 AND #5 AND #6 = #7
#7 NOT #8

Limiters: ENGLISH, ARTICLE

PubMed (267)
#1 (‘quality of life’)
“quality of life” [MeSH] OR “quality of living” [tiab] OR “life quality” [tiab] OR “welfare”[tiab] OR “standard of living” [tiab] OR “well-being” [tiab]

#2 (‘breast cancer’)
“breast cancer” [MeSH] OR “mammary cancer” [tiab] OR “breast carcinoma” [tiab]

#3 (‘upper limb’)
“upper limb” [tiab] OR “upper extremity” [tiab] OR “arm” [tiab] OR “hand” [tiab]

#4 (‘lymphedema’)
“lymphedema” [MeSH] OR “lymphoedema” [tiab] OR “lymphodema” [tiab]

#5 (‘questionnaire’)
“questionnaire” [tiab] OR “survey” [tiab] or “inquiry” [tiab] OR “question sheet” [tiab] OR “enquiry” [tiab]

#6 (‘measurement properties’) (Terwee et al, 2009)
(instrumentation[sh] OR methods[sh] OR “Validation Studies”[pt] OR “Comparative Study”[pt] OR “psychometrics”[MeSH] OR psychometr*[tiab] OR clinimetr*[tw] OR clinometr*[tw] OR “outcome assessment (health care)”[MeSH] OR “outcome assessment”[tiab] OR “outcome measure*”[tw] OR “observer variation”[MeSH] OR “observer variation”[tiab] OR “Health Status Indicators”[Mesh] OR “reproducibility of results”[MeSH] OR reproducib*[tiab] OR “discriminant analysis”[MeSH] OR reliab*[tiab] OR unreliab*[tiab] OR valid*[tiab] OR “coefficient of variation”[tiab] OR coefficient[tiab] OR homogeneity[tiab] OR homogeneous[tiab] OR “internal consistency”[tiab] OR (cronbach*[tiab] AND (alpha[tiab] OR alphas[tiab])) OR (item[tiab] AND (correlation*[tiab] OR selection*[tiab] OR reduction*[tiab])) OR agreement[tw] OR precision[tw] OR imprecision[tw] OR “precise values”[tw] OR test-retest[tiab] OR (test[tiab] AND retest[tiab]) OR (reliab*[tiab] AND (test[tiab] OR retest[tiab])) OR stability[tiab] OR interrater[tiab] OR inter-rater[tiab] OR intrarater[tiab] OR intra-rater[tiab] OR intertester[tiab] OR inter-tester[tiab] OR intratester[tiab] OR intra-tester[tiab] OR interobserver[tiab] OR inter-observer[tiab] OR intraobserver[tiab] OR intra-observer[tiab] OR intertechnician[tiab] OR inter-technician[tiab] OR intratechnician[tiab] OR intra-technician[tiab] OR interexaminer[tiab] OR inter-examiner[tiab] OR intraexaminer[tiab] OR intra-examiner[tiab] OR interassay[tiab] OR inter-assay[tiab] OR intraassay[tiab] OR intra-assay[tiab] OR interindividual[tiab] OR inter-individual[tiab] OR intraindividual[tiab] OR intra-individual[tiab] OR interparticipant[tiab] OR inter-participant[tiab] OR intraparticipant[tiab] OR intra-participant[tiab] OR kappa[tiab] OR kappa’s[tiab] OR kappas[tiab] OR repeatab*[tw] OR ((replicab*[tw] OR repeated[tw]) AND (measure[tw] OR measures[tw] OR findings[tw] OR result[tw] OR results[tw] OR test[tw] OR tests[tw])) OR generaliza*[tiab] OR generalisa*[tiab] OR concordance[tiab] OR (intraclass[tiab] AND correlation*[tiab]) OR discriminative[tiab] OR “known group”[tiab] OR “factor analysis”[tiab] OR “factor analyses”[tiab] OR “factor structure”[tiab] OR “factor structures”[tiab] OR dimension*[tiab] OR subscale*[tiab] OR (multitrait[tiab] AND scaling[tiab] AND (analysis[tiab] OR analyses[tiab])) OR “item discriminant”[tiab] OR “interscale correlation*”[tiab] OR error[tiab] OR errors[tiab] OR “individual variability”[tiab] OR “interval variability”[tiab] OR “rate variability”[tiab] OR (variability[tiab] AND (analysis[tiab] OR values[tiab])) OR (uncertainty[tiab] AND (measurement[tiab] OR measuring[tiab])) OR “standard error of measurement”[tiab] OR sensitiv*[tiab] OR responsive*[tiab] OR (limit[tiab] AND detection[tiab]) OR “minimal detectable concentration”[tiab] OR interpretab*[tiab] OR ((minimal[tiab] OR minimally[tiab] OR clinical[tiab] OR clinically[tiab]) AND (important[tiab] OR significant[tiab] OR detectable[tiab]) AND (change[tiab] OR difference[tiab])) OR (small*[tiab] AND (real[tiab] OR detectable[tiab]) AND (change[tiab] OR difference[tiab])) OR “meaningful change”[tiab] OR “ceiling effect”[tiab] OR “floor effect”[tiab] OR “Item response model”[tiab] OR IRT[tiab] OR Rasch[tiab] OR “Differential item functioning”[tiab] OR DIF[tiab] OR “computer adaptive testing”[tiab] OR “item bank”[tiab] OR “cross-cultural equivalence”[tiab])

#7 (‘exclusion filter’) (Terwee et al, 2009)
(“addresses”[Publication Type] OR “biography”[Publication Type] OR “case reports”[Publication Type] OR “comment”[Publication Type] OR “directory”[Publication Type] OR “editorial”[Publication Type] OR “festschrift”[Publication Type] OR “interview”[Publication Type] OR “lectures”[Publication Type] OR “legal cases”[Publication Type] OR “legislation”[Publication Type] OR “letter”[Publication Type] OR “news”[Publication Type] OR “newspaper article”[Publication Type] OR “patient education handout”[Publication Type] OR “popular works”[Publication Type] OR “congresses”[Publication Type] OR “consensus development conference”[Publication Type] OR “consensus development conference,

nih"[Publication Type] OR "practice guideline"[Publication Type]) NOT ("animals"[MeSH Terms] NOT "humans"[MeSH Terms])

Combination search
#1 AND #2 AND #4 AND #5 AND #6 = #7
#7 NOT #8

Additional filters: FULL TEXT, JOURNAL ARTICLE, VALIDATION STUDY, HUMANS, ENGLISH, FEMALES
